# Supplementary material for: Prevalence of Pathogenic Leptospira spp. in Non-Volant Small Mammals of Hutan Lipur Sekayu, Terengganu, Malaysia
Source: Pathogens. 2022 Nov 5;11(11):1300. doi: 10.3390/pathogens11111300 (PMC9693538; doi:10.3390/pathogens11111300)
Supplement: Supplementary file 1 [file pathogens-11-01300-s001.zip › pathogens-1987253-SI.pdf]

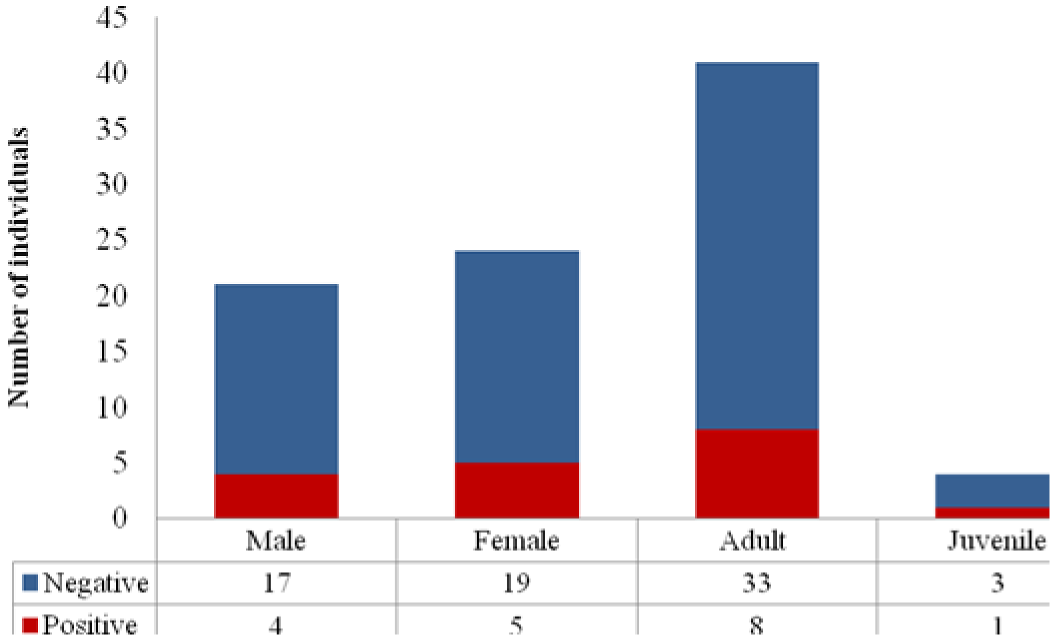

Supplementary material S1. Positive pathogenic *Leptospira* sp. infestation rate according to gender and age.
